# Supplementary material for: MetaRibo-Seq measures translation in microbiomes
Source: Nat Commun. 2020 Jun 29;11:3268. doi: 10.1038/s41467-020-17081-z (PMC7324362; doi:10.1038/s41467-020-17081-z)
Supplement: Supplementary file 10 — Supplementary Data 7 [file 41467_2020_17081_MOESM10_ESM.zip › File2/Confidence_High_Taxonomy/147174_out.krona.html]

Javascript must be enabled to view this page.

members
magnitude
magnitudeUnassigned
count
unassigned
taxon
rank

147174\_out

2

superkingdom
2
2

2
1239
phylum

186801
2
class

186802
2
order

2
186803
family

1164882
1
genus

467210
1

SRS144621\_contig\_number\_25567
species

genus
1
572511

species

SRS1041145\_contig\_number\_9057
1
33039
